# Real versus Simulated Mobile Phone Exposures in Experimental Studies

**DOI:** 10.1155/2015/607053

**Published:** 2015-08-05

**Authors:** Dimitris J. Panagopoulos, Olle Johansson, George L. Carlo

**Affiliations:** ^1^National Center for Scientific Research “Demokritos”, 60037 Athens, Greece; ^2^Department of Biology, University of Athens, 15784 Athens, Greece; ^3^Radiation and Environmental Biophysics Research Centre, 11143 Athens, Greece; ^4^Experimental Dermatology Unit, Department of Neuroscience, Karolinska Institute, 171 77 Stockholm, Sweden; ^5^The Science and Public Policy Institute, Institute for Healthful Adaptation, Falls Church, VA 22044, USA

## Abstract

We examined whether exposures to mobile phone radiation in biological/clinical experiments should be performed with real-life Electromagnetic Fields (EMFs) emitted by commercially available mobile phone handsets, instead of simulated EMFs emitted by generators or test phones. Real mobile phone emissions are constantly and unpredictably varying and thus are very different from simulated emissions which employ fixed parameters and no variability. This variability is an important parameter that makes real emissions more bioactive. Living organisms seem to have decreased defense against environmental stressors of high variability. While experimental studies employing simulated EMF-emissions present a strong inconsistency among their results with less than 50% of them reporting effects, studies employing real mobile phone exposures demonstrate an almost 100% consistency in showing adverse effects. This consistency is in agreement with studies showing association with brain tumors, symptoms of unwellness, and declines in animal populations. Average dosimetry in studies with real emissions can be reliable with increased number of field measurements, and variation in experimental outcomes due to exposure variability becomes less significant with increased number of experimental replications. We conclude that, in order for experimental findings to reflect reality, it is crucially important that exposures be performed by commercially available mobile phone handsets.

## 1. Introduction

Determination of realistic exposures from mobile phones and other wireless devices of modern telecommunications remains an important scientific challenge, especially since it is key to defining public health protection. The situation is further complicated by divergent results reported in the related literature that very well could be due to unrealistic exposure conditions, which in turn lead to ineffective and misdirected interventions.

The International Agency for Research on Cancer (IARC), while still classifying Radio Frequency (RF) Electromagnetic Fields (EMFs) as possibly carcinogenic, criticized and excluded from consideration experimental studies that used commercially available mobile phone handsets in exposing biological samples, as having “unreliable dosimetry” [1], without further scientific rationale. Similarly the Health Protection Agency (HPA) criticized this exposure methodology reporting that the exposure is “highly variable” with “lack of control” due to network reasons (number of subscribers each moment) and movement of the animals within the vials/boxes in case of freely moving animals but recognizes that restriction of the animals during the exposures will result in additional stress. Their critique recommended that exposures should be performed by devices or handsets set to produce emissions at fixed frequency and output power by use of engineering or hardware controls [2]. In both reports the criticisms were based on the fact that real mobile phone emissions always include significant variations in their intensity, frequency, and other parameters, especially in the near-field of the antenna.

But billions of mobile phone users are daily exposed for increasing periods to real emissions from their handsets in the near-field of the antenna in contact with their ears/bodies, not to any simulated emissions with fixed parameters. Is it then scientifically correct to study the effects of a “highly variable” field by using fields with fixed parameters? In our opinion, it is not, especially in the case when the varying nature of the field seems to be an important reason for its increased biological activity.

The aim of the present study is to review biological and clinical experimental studies on mobile phone radiation effects which have employed exposures with real mobile phone emissions, as opposed to the mainstream studies which employ simulated mobile phone emissions produced by generators or test phones, and seek an explanation for the divergent results reported in the literature. In case that we find a significant conflict in the results between the two types of experimental exposures (real versus simulated), our aim is to attempt giving an explanation based on the differences between the two types of EMF-emissions.

We note that the issue of the present study applies also for every other type of RF/microwave emitting devices used in modern telecommunications, such as Internet connection wireless devices and local wireless networks (Wi-Fi), domestic cordless phones (DECT, Digitally Enhanced Cordless Technology), and baby monitors. The emissions from all these devices, although differing in specific frequencies and modulation types, are very similar. The reason that we concentrate on studies with mobile phone radiation (either real or simulated) is only the fact that they constitute the vast majority of the published studies testing the biological activity of RF/microwave EMFs.

## 2. Adaptation of Living Organisms to EMFs

Living organisms have been constantly exposed throughout evolution to terrestrial static electric and magnetic fields of average intensities ~130 V/m and ~0.5 G, respectively. While no adverse health effects are connected with usual exposure to these natural ambient fields, variations in their intensities on the order of 20% during “magnetic storms” or “geomagnetic pulsations” due to changes in solar activity with an average periodicity of about 11 years are connected with increased rates of animal/human health incidents, including nervous and psychic diseases, hypertensive crises, heart attacks, cerebral accidents, and mortality [3, 4].

It is clear that living organisms perceive EMFs as environmental stressors [4–7]. But since man-made EMFs constitute a very new stressor for living organisms within the billions of years of biological evolution, the cells have not developed defensive mechanisms, for example, special genes to be activated for protection against electromagnetic stress of man-made EMFs. This can be the reason why in response to man-made EMFs cells are found to activate heat-shock genes and produce heat-shock proteins very rapidly (within minutes) and at a much higher rate than for heat itself [6]. It seems to be for the same reason that mobile phone radiation is found to induce DNA damage and cell death in insect reproductive cells at a higher degree than other types of external stressors examined before like food deprivation or chemicals [8–10]. Thus it appears that cells are much more sensitive to man-made EMFs than to other types of stress previously experienced by living organisms such as heat, cold, starvation, or chemicals. But repetitive stress leading to continuous expression of heat-shock genes or DNA damage may lead to cancer [1, 11].

One reason for the increased biological activity of man-made EMFs can be that cells/organisms adapt more easily to any external stressor, and to EMFs, when this stressor is not of significantly varying type, in other words when its parameters are kept constant or vary only slightly. Since living organisms do not have defense mechanisms against variations on the order of 20% of natural EMFs as explained above, it is realistic to expect that they do not have innate defenses against unnatural (man-made) EMFs, which are mostly not static but varying (alternating, pulsed, modulated fields, including simultaneously several different frequencies, etc.) and totally polarized in contrast to natural EMFs. [We note that even though the polarities and intensities of the static terrestrial electric and magnetic fields do not change significantly (except during specific periods as explained) there are always small changes and local variations in the direction of the field lines that make these natural static fields only partially and never totally polarized [3, 4]. This is in contrast to all man-made EMFs which are totally and invariantly polarized due to the invariant geometry of their electric circuits.]

Indeed, pulsed or modulated electromagnetic signals (radiation) are found in numerous studies published since the midseventies to be more bioactive than continuous signals of identical other parameters (intensity, frequency, duration, waveform, etc.) [12–24]. Moreover, intermittent exposure to mobile phone radiation (real or simulated) with short intermittence durations (which makes the field even more variable) is repeatedly found to be more bioactive than the corresponding continuous exposure [25, 26]. This experimental evidence further supports the argument that the more complicated and variable the field/stressor is, the more difficult it is for a living organism to adapt to it.

## 3. The Increased Variability of EMFs Emitted by Mobile Telephony Antennas

All types of digital mobile telephony radiation, except for their RF carrier signal, employ Extremely Low Frequencies (ELF) necessary for the modulation and for increasing the capacity of transmitted information by pulsing the signal. The combination of the RF carrier and the ELF pulsing frequencies has been found to be more bioactive than the RF carrier alone [16, 21]. Moreover, according to a plausible suggested mechanism [27], (a) the ELF frequencies included in any pulsed or modulated RF signal are more responsible for the biological effects, (b) changes in field intensity play a major role, and (c) the pulsing of the signal makes it twice more bioactive. A constant carrier RF wave modulated by a constant ELF field can certainly be simulated but this is not the case in real mobile telephony signals, in which both the carrier and the modulation are constantly and unpredictably varying in intensity, frequency, and waveform during a phone-conversation [7, 28–30].

The intensity of radiation varies significantly each moment during a usual phone-conversation depending on signal reception, number of subscribers sharing the frequency band each moment, air conductivity, location within the wireless infrastructure, presence of objects and metallic surfaces, “speaking” versus “nonspeaking” mode, and so forth. These variations are much larger than 20% of the average signal intensity (as opposed to the periodical variations in the terrestrial fields known to cause health effects). Moreover the phase of the carrier signal varies continuously during a phone-conversation, and the RF frequency constantly changes between different available frequency channels, especially in third generation (3G) radiation. The wave shape is also constantly changing depending on how the changing information transmitted each moment modulates the carrier wave. Thus, the parameters of this radiation change constantly and unpredictably each moment and large, sudden, unpredictable variations in the emitted EMF/radiation take place constantly during a usual phone-conversation. The more the amount of carried information is increased (by adding text, speech, pictures, music, video, internet, etc.) in more recent phone generations (G)/types (2G, 3G, 4G, etc.), the more complicated and unpredictably varying the cell phone signals become [2, 7, 28–30].

Thus, real digital mobile phone (and other wireless communication devices) emissions change constantly and unpredictably. As a consequence, living organisms cannot adapt to such a highly varying type of stress. Moreover, due to the unpredictably varying type of the real emissions, it is impossible to simulate them by EMFs of fixed parameters.

## 4. Real Exposure Studies as Opposed to Studies with Simulated Exposures

A significant number of studies have already been published which employed commercially available mobile phones during connection (“talk”, “listen”, or “call” modes) for exposure to a wide variety of animals (including humans)/biological samples, including* Drosophila* [6, 8, 26, 31–37], ants [38], chicken eggs [39], quails [40], human sperm* in vitro* [41, 42], human volunteers* in vivo* [43–52], mice or rats or guinea-pigs or rabbits* in vivo* [53–69], mouse cells* in vitro* [70], bees [71–73], protozoa [74], and even purified proteins* in vitro* [75]. An impressive percentage (95.8%) of these studies (46 out of 48 studies with real-life exposures) have recorded significant adverse biological or clinical effects, ranging from loss of orientation, kinetic changes, and behavioral or electroencephalographic (EEG) changes to decrease in male and female reproductive capacity, reproductive declines, molecular changes, changes in enzymatic activity, DNA damage and cell death, and histopathological changes in the brain. It was found that during “talk” mode (voice modulation) the exposure is significantly more bioactive than during “listen” mode due to the voice modulation and associated increased intensity of the emissions [7, 31]. From the remaining two studies, one reported no effect [55] and one reported an increase in short-term memory of children [47] which we do not count as an adverse effect although it may be.

On the contrary, more than 50% of the studies performed with simulated signals have showed no effects [1, 2, 76], even though several recent review studies suggest an overall predominance of studies showing effects regardless of real or simulated exposures [7, 77–80]. A recent meta-analysis of 88 studies published during 1990–2011 investigating genetic damage in human cells from RF radiation, 87 of which did not employ real telecommunication EMFs, reported no overall association with genotoxicity [81].

Although we may have missed a few more studies with real mobile phone exposures, it becomes evident that there is a strong conflict between the overall results of studies performed with real mobile phone emissions and the overall results of studies with simulated emissions from generators and “test” phones. Moreover, while within the group of studies with simulated emissions there is also a conflict between studies that find effects and studies that do not, the group of studies with real exposures demonstrates an impressive consistency in showing effects almost at 100%. Moreover, this impressive consistency is corroborated by increasing epidemiological evidence, especially during the last years, for an association between (real-life) mobile phone use and brain tumors [82–84], by statistical studies reporting symptoms of unwellness among people residing around mobile telephony base station antennas or among mobile phone users [85–90], and by open field studies reporting declines in bird and amphibian populations around mobile telephony base station antennas [91–95].

This apparent consistency of results in the laboratory studies with real emissions and their additional corroboration with recent epidemiological/statistical and open field studies' evidence seems to be unnoticed by health agencies and public health authorities which simply disregard these studies despite their important findings which imply the urgent establishment of much more stringent exposure limits than the current ones [96].

Although in most studies employing real mobile phone emissions the biological samples were exposed in close proximity (within the near-field up to approximately 5 cm) with the mobile phone handset, in several studies the samples/animals were exposed at greater distances in the far-field up to 1 m [32, 34, 35, 39, 51, 53, 56–58] where the intensity variations are much smaller and the dosimetry is absolutely “reliable” as is generally accepted for far-field antenna measurements [97]. In one of these studies it was found that at 20–30 cm distance from the mobile phone the biological effect (DNA damage) was even more intense than at zero distance [32].

A mobile phone antenna's near-field extends to a distance of 5.2 or 2.6 cm, for 900 or 1800 MHz, respectively (most commonly employed carrier frequencies in 2G mobile telephony radiation), according to the relation *r* = *λ*/2*π*, (*r* is the distance of near-field far limit from the antenna when the length of the antenna is smaller than the wavelength *λ* of the emitted radiation) [98].

In studies with real mobile phone emissions investigating the dependence of observed effects on dose (radiation intensity and/or exposure duration) [8, 31–35, 39, 40, 62], the effects have been found to be dose dependent. The dependence on dose was in most cases nonlinear, although in two studies the dependence of certain effects on exposure duration was approximating linearity [35, 62].

The results of experiments with real-life (variable) mobile phone EMFs are indeed not identically reproducible, since between successive exposures at any specific location the exact characteristics of the emitted signal are always different. But the average field values over a few minutes' (or more) period are close to each other, and thus the results of different replicate experiments with real emissions as the independent variable, although not identical quantitatively, are qualitatively similar. Statistical significance in the results can be increased by increasing the number of experimental replications while keeping rigorous control of all other parameters (animal/sample conditions, temperature, humidity, light, stray EMFs within the lab, etc.). Then, as the number of replications increases, field variability becomes less significant [99].

## 5. Discussion

In the present study we showed that the percentages of positive results differ significantly between studies with real mobile phone exposures and studies with simulated exposures, regardless of biological samples or other procedure details. The basic difference between real and simulated mobile telephony EMFs is the inherent significant variability of the first which we believe is the reason for the strong divergence in the experimental results.

In spite of the criticism on the studies employing real exposures by health agencies [1, 2] (the different aspects of which we extensively addressed) and the consequent difficulty in the publication process, the number of studies with real mobile phone emissions is increasing rapidly in the peer-reviewed literature, especially during the last years. An increasing number of scientists realize that real exposures by commercially available mobile phone handsets are the only way to represent conditions experienced by users in real-life, since they are very different and considerably more bioactive than the exposures made by simulated fields.

Any variability in the field and correspondingly in the dosimetry does not change the fact that people are actually exposed daily for increasing periods to this “highly variable” field in contact with their heads/bodies and at different distances. The presented scientific data show that this constant variation in the field makes it considerably more active biologically.

In order to have a measure of this variability, RF and ELF measurements of average intensity ± standard deviation (SD) of the emitted real EMFs should be included in the studies, in addition to the Specific Absorption Rate (*SAR*) information supplied by the manufacturer (referring to a simulated human head [100]). With increasing number of measurements the SD decreases enough for the dosimetry to be judged as reliable [8, 26, 31–36, 99].

If we accepted that the real EMFs emitted by commercially available mobile phones are so much variable and their dosimetry is so much unreliable that the studies employing real EMF-emissions are not to be taken into account because of “unknown” dosimetry, then these devices should not be approved by the public authorities to be available in the market, since unpredictable unmeasurable signal changes can result in unpredictable biological alterations. Once these devices are approved for the market (a fact that we do not challenge) the definition of the exposure is the* exposure to a user's head during a usual phone-conversation*, and this, in our opinion, should be enough for the studies to be taken into account by health agencies and authorities. Nevertheless, the measurements of the emitted EMFs suggested above are important to better quantify real-life exposures, in addition to verifying that the average emissions by the handsets used in the experiments do not transcend the existing limits [96].

It is useful to create simulations in order to study in the lab conditions of specific environments which are not accessible for laboratory work (outer space, underwater high depths, etc.). The simulations in such cases should be as close as possible to the real conditions. However, using nonrealistic simulations, especially when real conditions are easily accessible to be studied in the lab with well-controlled other parameters, is, in our opinion, a serious scientific flaw that is pervading the mobile phone bioeffects literature. The employment of simplified nonrealistic simulations may be useful for specific purposes, for example, to study what the effects would be if the signal characteristics were different, in order to improve them.

Experiments comparing the biological activity between real and simulated mobile telephony EMFs with similar average parameter values should urgently be conducted in order to test the validity of our presented arguments. Studies performed with simulated fields/exposures, especially those that did not show any effects, should, in our opinion, be repeated with real exposures of similar average signal parameters while keeping all the remaining experimental variables identical. In case that these experiments verify our arguments, health agencies should immediately revise their guidelines in regard to which studies should be considered most important and on whether the available data are indeed conflicting or not. Moreover, according to the precautionary principle, the existing exposure criteria should drastically be revised, since the effects reported in all studies with real mobile phone emissions have been recorded with EMF-intensities well below (up to thousands of times below) the existing exposure limits [8, 26, 31–75, 96].

Without account for real exposure parameters, studies suffer from imprecision that likely biases results toward null hypotheses, increasing the probability that true health risks among consumers are being missed. Simulated signals with fixed parameters bear little, if any, resemblance to what mobile phone users actually experience, even when they employ combinations of simulated signals [101–103].

In order for the biological/clinical studies testing the bioactivity of mobile telephony radiation to account for real conditions, we conclude that exposures should be performed by real EMFs as these are emitted by commercially available mobile phones. The same holds for experiments with other types of EMFs employed in modern telecommunication systems such as DECT phones and Wi-Fi. In addition to that, simulated emissions may be used to study, for example, the effects of separate parameters of the real EMFs, but in no way should simulated emissions substitute the real ones.

As the scientific database regarding the biological effects of EMFs emitted by modern telecommunications continues to grow, it is important for experimental study designs to grow in rigor and provide a more informed basis for interpretation. One important step is to employ real-life exposures.

To investigate the biological/health effects from a widely accessible device exposing daily billions of humans we should not try to simulate the device but simply use the device itself. In particular, we should not try to simulate its real varying emissions with totally unrealistic invariant ones. This is a serious scientific flaw that may lead to totally devious results with enormous adverse consequences for public health.
